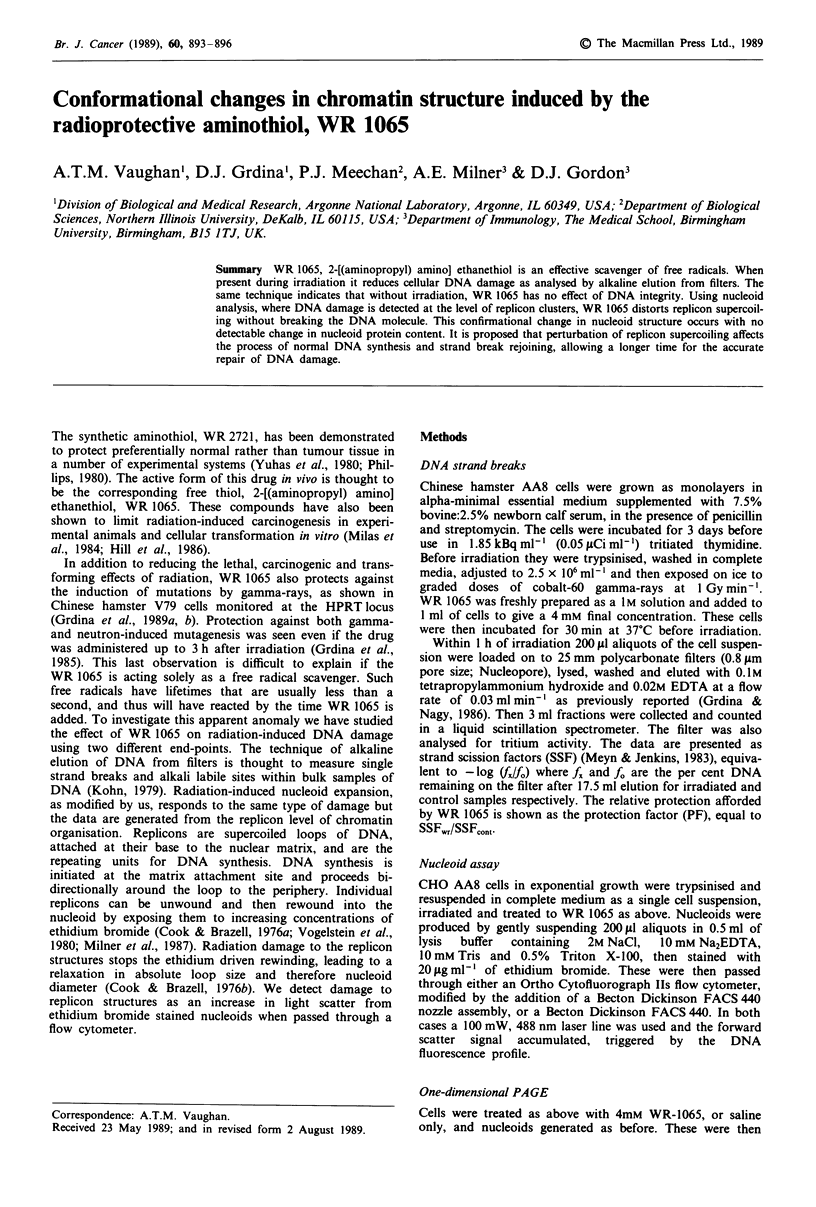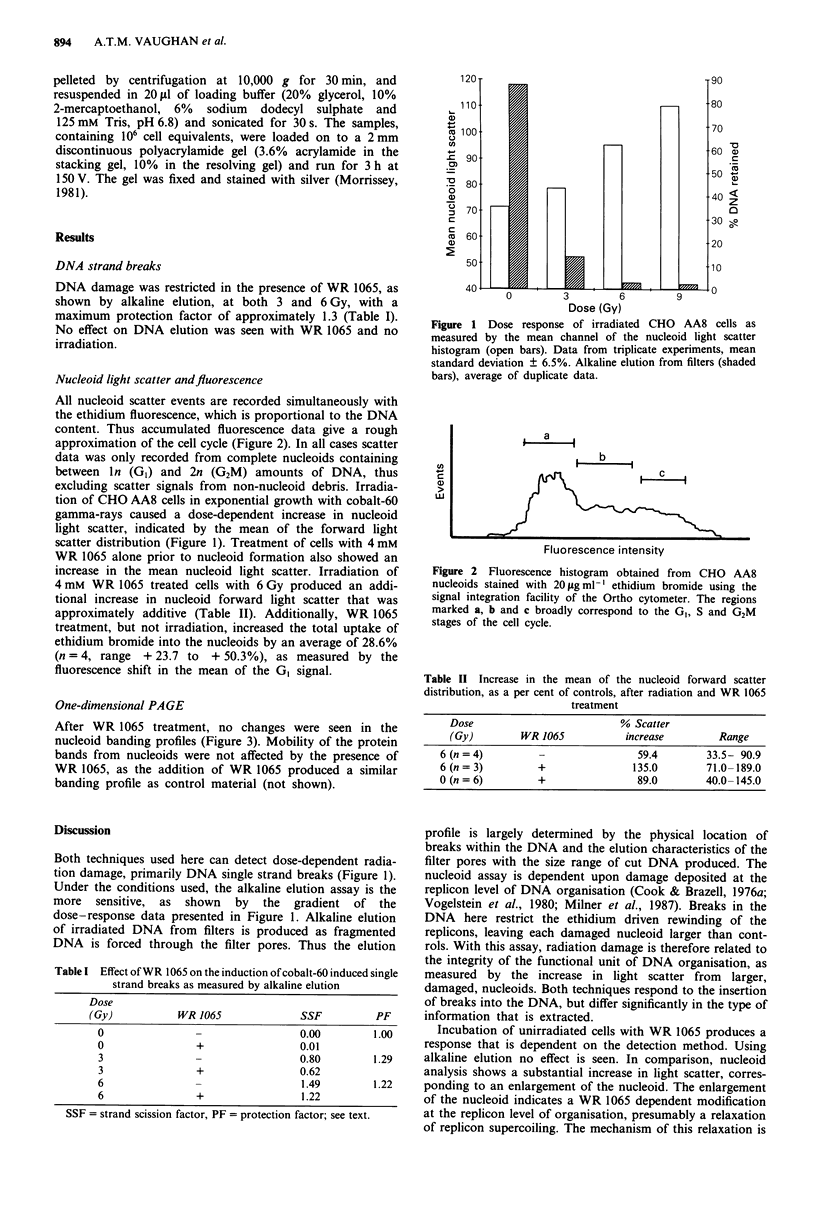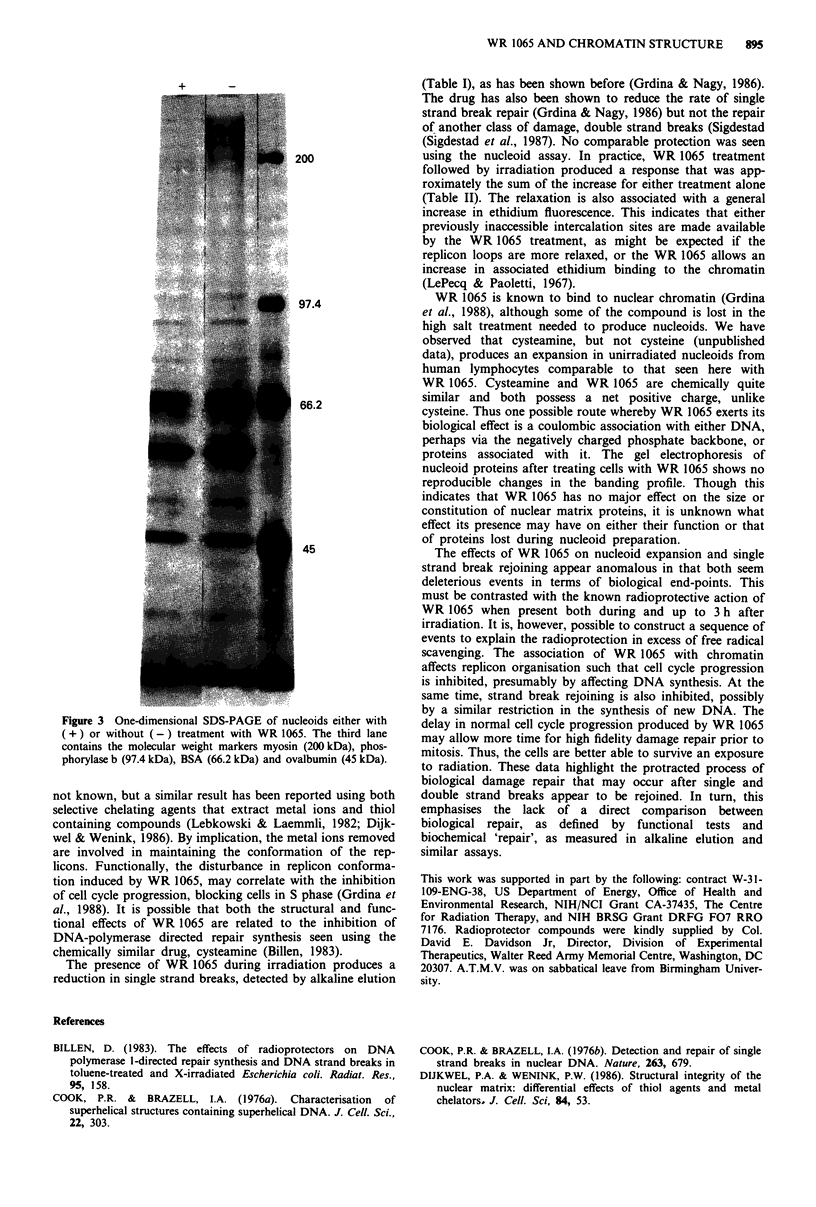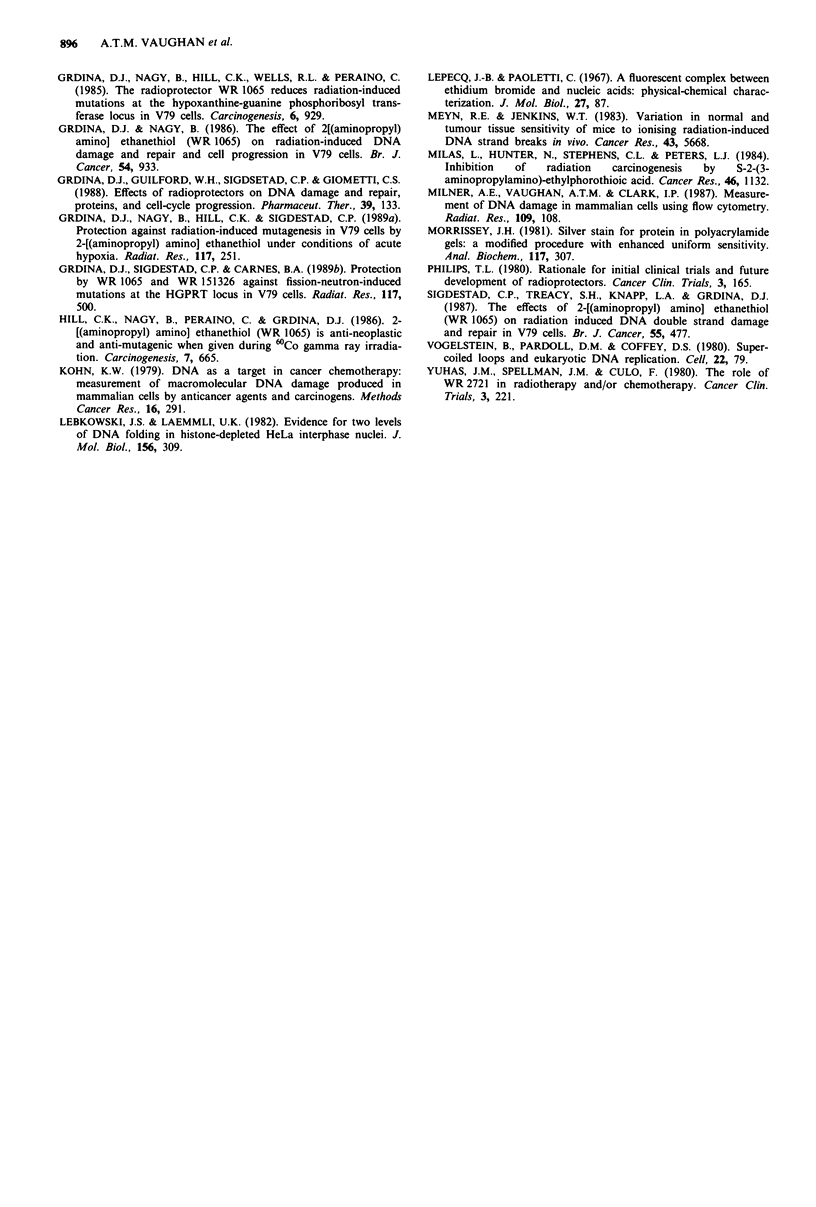# Conformational changes in chromatin structure induced by the radioprotective aminothiol, WR 1065.

**DOI:** 10.1038/bjc.1989.386

**Published:** 1989-12

**Authors:** A. T. Vaughan, D. J. Grdina, P. J. Meechan, A. E. Milner, D. J. Gordon

**Affiliations:** Division of Biological and Medical Research, Argonne National Laboratory, IL 60349.

## Abstract

**Images:**


Br. J. Cancer (1989), 60, 893–896 © The Macmillan Press Ltd., 1989

### Conformational changes in chromatin structure induced by the radioprotective aminothiol, WR 1065

A.T.M. Vaughan[1], D.J. Grdina[1], P.J. Meechan[2], A.E. Milner[3] & D.J. Gordon[3]

[1]*Division of Biological and Medical Research, Argonne National Laboratory, Argonne, IL 60349, USA;* [2]*Department of Biological Sciences, Northern Illinois University, DeKalb, IL 60115, USA;* [3]*Department of Immunology, The Medical School, Birmingham University, Birmingham, B15 1TJ, UK.*

**Summary** WR 1065, 2-[(aminopropyl) amino] ethanethiol is an effective scavenger of free radicals. When present during irradiation it reduces cellular DNA damage as analysed by alkaline elution from filters. The same technique indicates that without irradiation, WR 1065 has no effect of DNA integrity. Using nucleoid analysis, where DNA damage is detected at the level of replicon clusters, WR 1065 distorts replicon supercoiling without breaking the DNA molecule. This confirmational change in nucleoid structure occurs with no detectable change in nucleoid protein content. It is proposed that perturbation of replicon supercoiling affects the process of normal DNA synthesis and strand break rejoining, allowing a longer time for the accurate repair of DNA damage.

The synthetic aminothiol, WR 2721, has been demonstrated to protect preferentially normal rather than tumour tissue in a number of experimental systems (Yuhas *et al.*, 1980; Phillips, 1980). The active form of this drug *in vivo* is thought to be the corresponding free thiol, 2-[(aminopropyl) amino] ethanethiol, WR 1065. These compounds have also been shown to limit radiation-induced carcinogenesis in experimental animals and cellular transformation *in vitro* (Milas *et al.*, 1984; Hill *et al.*, 1986).

In addition to reducing the lethal, carcinogenic and transforming effects of radiation, WR 1065 also protects against the induction of mutations by gamma-rays, as shown in Chinese hamster V79 cells monitored at the HPRT locus (Grdina *et al.*, 1989*a*, *b*). Protection against both gamma- and neutron-induced mutagenesis was seen even if the drug was administered up to 3 h after irradiation (Grdina *et al.*, 1985). This last observation is difficult to explain if the WR 1065 is acting solely as a free radical scavenger. Such free radicals have lifetimes that are usually less than a second, and thus will have reacted by the time WR 1065 is added. To investigate this apparent anomaly we have studied the effect of WR 1065 on radiation-induced DNA damage using two different end-points. The technique of alkaline elution of DNA from filters is thought to measure single strand breaks and alkali labile sites within bulk samples of DNA (Kohn, 1979). Radiation-induced nucleoid expansion, as modified by us, responds to the same type of damage but the data are generated from the replicon level of chromatin organisation. Replicons are supercoiled loops of DNA, attached at their base to the nuclear matrix, and are the repeating units for DNA synthesis. DNA synthesis is initiated at the matrix attachment site and proceeds bi-directionally around the loop to the periphery. Individual replicons can be unwound and then rewound into the nucleoid by exposing them to increasing concentrations of ethidium bromide (Cook & Brazell, 1976*a*; Vogelstein *et al.*, 1980; Milner *et al.*, 1987). Radiation damage to the replicon structures stops the ethidium driven rewinding, leading to a relaxation in absolute loop size and therefore nucleoid diameter (Cook & Brazell, 1976*b*). We detect damage to replicon structures as an increase in light scatter from ethidium bromide stained nucleoids when passed through a flow cytometer.

Correspondence: A.T.M. Vaughan.
Received 23 May 1989; and in revised form 2 August 1989.

### Methods

### *DNA strand breaks*

Chinese hamster AA8 cells were grown as monolayers in alpha-minimal essential medium supplemented with 7.5% bovine:2.5% newborn calf serum, in the presence of penicillin and streptomycin. The cells were incubated for 3 days before use in 1.85 kBq $ml^{-1}$ (0.05 $\mu$Ci $ml^{-1}$) tritiated thymidine. Before irradiation they were trypsinised, washed in complete media, adjusted to $2.5 \times 10^6$ $ml^{-1}$ and then exposed on ice to graded doses of cobalt-60 gamma-rays at 1 Gy $min^{-1}$. WR 1065 was freshly prepared as a 1 M solution and added to 1 ml of cells to give a 4 mM final concentration. These cells were then incubated for 30 min at 37°C before irradiation.

Within 1 h of irradiation 200 $\mu$l aliquots of the cell suspension were loaded on to 25 mm polycarbonate filters (0.8 $\mu$m pore size; Nucleopore), lysed, washed and eluted with 0.1 M tetrapropylammonium hydroxide and 0.02 M EDTA at a flow rate of 0.03 ml $min^{-1}$ as previously reported (Grdina & Nagy, 1986). Then 3 ml fractions were collected and counted in a liquid scintillation spectrometer. The filter was also analysed for tritium activity. The data are presented as strand scission factors (SSF) (Meyn & Jenkins, 1983), equivalent to $-\log(f_x/f_o)$ where $f_x$ and $f_o$ are the per cent DNA remaining on the filter after 17.5 ml elution for irradiated and control samples respectively. The relative protection afforded by WR 1065 is shown as the protection factor (PF), equal to $SSF_{wr}/SSF_{cont}$.

### *Nucleoid assay*

CHO AA8 cells in exponential growth were trypsinised and resuspended in complete medium as a single cell suspension, irradiated and treated to WR 1065 as above. Nucleoids were produced by gently suspending 200 $\mu$l aliquots in 0.5 ml of lysis buffer containing 2 M NaCl, 10 mM $Na_2EDTA$, 10 mM Tris and 0.5% Triton X-100, then stained with 20 $\mu$g $ml^{-1}$ of ethidium bromide. These were then passed through either an Ortho Cytofluorograph IIs flow cytometer, modified by the addition of a Becton Dickinson FACS 440 nozzle assembly, or a Becton Dickinson FACS 440. In both cases a 100 mW, 488 nm laser line was used and the forward scatter signal accumulated, triggered by the DNA fluorescence profile.

### *One-dimensional PAGE*

Cells were treated as above with 4mM WR-1065, or saline only, and nucleoids generated as before. These were then pelleted by centrifugation at 10,000 *g* for 30 min, and resuspended in 20 µl of loading buffer (20% glycerol, 10% 2-mercaptoethanol, 6% sodium dodecyl sulphate and 125 mM Tris, pH 6.8) and sonicated for 30 s. The samples, containing $10^6$ cell equivalents, were loaded on to a 2 mm discontinuous polyacrylamide gel (3.6% acrylamide in the stacking gel, 10% in the resolving gel) and run for 3 h at 150 V. The gel was fixed and stained with silver (Morrissey, 1981).

### Results

### *DNA strand breaks*

DNA damage was restricted in the presence of WR 1065, as shown by alkaline elution, at both 3 and 6 Gy, with a maximum protection factor of approximately 1.3 (Table I). No effect on DNA elution was seen with WR 1065 and no irradiation.

### *Nucleoid light scatter and fluorescence*

All nucleoid scatter events are recorded simultaneously with the ethidium fluorescence, which is proportional to the DNA content. Thus accumulated fluorescence data give a rough approximation of the cell cycle (Figure 2). In all cases scatter data was only recorded from complete nucleoids containing between 1*n* ($G_1$) and 2*n* ($G_2M$) amounts of DNA, thus excluding scatter signals from non-nucleoid debris. Irradiation of CHO AA8 cells in exponential growth with cobalt-60 gamma-rays caused a dose-dependent increase in nucleoid light scatter, indicated by the mean of the forward light scatter distribution (Figure 1). Treatment of cells with 4 mM WR 1065 alone prior to nucleoid formation also showed an increase in the mean nucleoid light scatter. Irradiation of 4 mM WR 1065 treated cells with 6 Gy produced an additional increase in nucleoid forward light scatter that was approximately additive (Table II). Additionally, WR 1065 treatment, but not irradiation, increased the total uptake of ethidium bromide into the nucleoids by an average of 28.6% ($n = 4$, range +23.7 to +50.3%), as measured by the fluorescence shift in the mean of the $G_1$ signal.

### *One-dimensional PAGE*

After WR 1065 treatment, no changes were seen in the nucleoid banding profiles (Figure 3). Mobility of the protein bands from nucleoids were not affected by the presence of WR 1065, as the addition of WR 1065 produced a similar banding profile as control material (not shown).

**Figure 1** Dose response of irradiated CHO AA8 cells as measured by the mean channel of the nucleoid light scatter histogram (open bars). Data from triplicate experiments, mean standard deviation ± 6.5%. Alkaline elution from filters (shaded bars), average of duplicate data.

**Figure 2** Fluorescence histogram obtained from CHO AA8 nucleoids stained with 20 µg ml$^{-1}$ ethidium bromide using the signal integration facility of the Ortho cytometer. The regions marked **a**, **b** and **c** broadly correspond to the $G_1$, S and $G_2M$ stages of the cell cycle.

**Table II** Increase in the mean of the nucleoid forward scatter distribution, as a per cent of controls, after radiation and WR 1065 treatment

| *Dose (Gy)* | *WR 1065* | *% Scatter increase* | *Range* |
|---|---|---|---|
| 6 ($n = 4$) | – | 59.4 | 33.5– 90.9 |
| 6 ($n = 3$) | + | 135.0 | 71.0–189.0 |
| 0 ($n = 6$) | + | 89.0 | 40.0–145.0 |

### Discussion

Both techniques used here can detect dose-dependent radiation damage, primarily DNA single strand breaks (Figure 1). Under the conditions used, the alkaline elution assay is the more sensitive, as shown by the gradient of the dose–response data presented in Figure 1. Alkaline elution of irradiated DNA from filters is produced as fragmented DNA is forced through the filter pores. Thus the elution profile is largely determined by the physical location of breaks within the DNA and the elution characteristics of the filter pores with the size range of cut DNA produced. The nucleoid assay is dependent upon damage deposited at the replicon level of DNA organisation (Cook & Brazell, 1976*a*; Vogelstein *et al.*, 1980; Milner *et al.*, 1987). Breaks in the DNA here restrict the ethidium driven rewinding of the replicons, leaving each damaged nucleoid larger than controls. With this assay, radiation damage is therefore related to the integrity of the functional unit of DNA organisation, as measured by the increase in light scatter from larger, damaged, nucleoids. Both techniques respond to the insertion of breaks into the DNA, but differ significantly in the type of information that is extracted.

**Table I** Effect of WR 1065 on the induction of cobalt-60 induced single strand breaks as measured by alkaline elution

| *Dose (Gy)* | *WR 1065* | *SSF* | *PF* |
|---|---|---|---|
| 0 | – | 0.00 | 1.00 |
| 0 | + | 0.01 | |
| 3 | – | 0.80 | 1.29 |
| 3 | + | 0.62 | |
| 6 | – | 1.49 | 1.22 |
| 6 | + | 1.22 | |

SSF = strand scission factor, PF = protection factor; see text.

Incubation of unirradiated cells with WR 1065 produces a response that is dependent on the detection method. Using alkaline elution no effect is seen. In comparison, nucleoid analysis shows a substantial increase in light scatter, corresponding to an enlargement of the nucleoid. The enlargement of the nucleoid indicates a WR 1065 dependent modification at the replicon level of organisation, presumably a relaxation of replicon supercoiling. The mechanism of this relaxation is

**Figure 3** One-dimensional SDS-PAGE of nucleoids either with ( + ) or without ( − ) treatment with WR 1065. The third lane contains the molecular weight markers myosin (200 kDa), phosphorylase b (97.4 kDa), BSA (66.2 kDa) and ovalbumin (45 kDa).

not known, but a similar result has been reported using both selective chelating agents that extract metal ions and thiol containing compounds (Lebkowski & Laemmli, 1982; Dijkwel & Wenink, 1986). By implication, the metal ions removed are involved in maintaining the conformation of the replicons. Functionally, the disturbance in replicon conformation induced by WR 1065, may correlate with the inhibition of cell cycle progression, blocking cells in S phase (Grdina *et al.*, 1988). It is possible that both the structural and functional effects of WR 1065 are related to the inhibition of DNA-polymerase directed repair synthesis seen using the chemically similar drug, cysteamine (Billen, 1983).

The presence of WR 1065 during irradiation produces a reduction in single strand breaks, detected by alkaline elution (Table I), as has been shown before (Grdina & Nagy, 1986). The drug has also been shown to reduce the rate of single strand break repair (Grdina & Nagy, 1986) but not the repair of another class of damage, double strand breaks (Sigdestad (Sigdestad *et al.*, 1987). No comparable protection was seen using the nucleoid assay. In practice, WR 1065 treatment followed by irradiation produced a response that was approximately the sum of the increase for either treatment alone (Table II). The relaxation is also associated with a general increase in ethidium fluorescence. This indicates that either previously inaccessible intercalation sites are made available by the WR 1065 treatment, as might be expected if the replicon loops are more relaxed, or the WR 1065 allows an increase in associated ethidium binding to the chromatin (LePecq & Paoletti, 1967).

WR 1065 is known to bind to nuclear chromatin (Grdina *et al.*, 1988), although some of the compound is lost in the high salt treatment needed to produce nucleoids. We have observed that cysteamine, but not cysteine (unpublished data), produces an expansion in unirradiated nucleoids from human lymphocytes comparable to that seen here with WR 1065. Cysteamine and WR 1065 are chemically quite similar and both possess a net positive charge, unlike cysteine. Thus one possible route whereby WR 1065 exerts its biological effect is a coulombic association with either DNA, perhaps via the negatively charged phosphate backbone, or proteins associated with it. The gel electrophoresis of nucleoid proteins after treating cells with WR 1065 shows no reproducible changes in the banding profile. Though this indicates that WR 1065 has no major effect on the size or constitution of nuclear matrix proteins, it is unknown what effect its presence may have on either their function or that of proteins lost during nucleoid preparation.

The effects of WR 1065 on nucleoid expansion and single strand break rejoining appear anomalous in that both seem deleterious events in terms of biological end-points. This must be contrasted with the known radioprotective action of WR 1065 when present both during and up to 3 h after irradiation. It is, however, possible to construct a sequence of events to explain the radioprotection in excess of free radical scavenging. The association of WR 1065 with chromatin affects replicon organisation such that cell cycle progression is inhibited, presumably by affecting DNA synthesis. At the same time, strand break rejoining is also inhibited, possibly by a similar restriction in the synthesis of new DNA. The delay in normal cell cycle progression produced by WR 1065 may allow more time for high fidelity damage repair prior to mitosis. Thus, the cells are better able to survive an exposure to radiation. These data highlight the protracted process of biological damage repair that may occur after single and double strand breaks appear to be rejoined. In turn, this emphasises the lack of a direct comparison between biological repair, as defined by functional tests and biochemical 'repair', as measured in alkaline elution and similar assays.

This work was supported in part by the following: contract W-31-109-ENG-38, US Department of Energy, Office of Health and Environmental Research, NIH/NCI Grant CA-37435, The Centre for Radiation Therapy, and NIH BRSG Grant DRFG FO7 RRO 7176. Radioprotector compounds were kindly supplied by Col. David E. Davidson Jr, Director, Division of Experimental Therapeutics, Walter Reed Army Memorial Centre, Washington, DC 20307. A.T.M.V. was on sabbatical leave from Birmingham University.

### References

BILLEN, D. (1983). The effects of radioprotectors on DNA polymerase 1-directed repair synthesis and DNA strand breaks in toluene-treated and X-irradiated *Escherichia coli*. *Radiat. Res.*, **95**, 158.

COOK, P.R. & BRAZELL, I.A. (1976*a*). Characterisation of superhelical structures containing superhelical DNA. *J. Cell. Sci.*, **22**, 303.

COOK, P.R. & BRAZELL, I.A. (1976*b*). Detection and repair of single strand breaks in nuclear DNA. *Nature*, **263**, 679.

DIJKWEL, P.A. & WENINK, P.W. (1986). Structural integrity of the nuclear matrix: differential effects of thiol agents and metal chelators. *J. Cell. Sci*, **84**, 53.

GRDINA, D.J., NAGY, B., HILL, C.K., WELLS, R.L. & PERAINO, C. (1985). The radioprotector WR 1065 reduces radiation-induced mutations at the hypoxanthine-guanine phosphoribosyl transferase locus in V79 cells. *Carcinogenesis*, **6**, 929.

GRDINA, D.J. & NAGY, B. (1986). The effect of 2[(aminopropyl) amino] ethanethiol (WR 1065) on radiation-induced DNA damage and repair and cell progression in V79 cells. *Br. J. Cancer*, **54**, 933.

GRDINA, D.J., GUILFORD, W.H., SIGDSETAD, C.P. & GIOMETTI, C.S. (1988). Effects of radioprotectors on DNA damage and repair, proteins, and cell-cycle progression. *Pharmaceut. Ther.*, **39**, 133.

GRDINA, D.J., NAGY, B., HILL, C.K. & SIGDESTAD, C.P. (1989*a*). Protection against radiation-induced mutagenesis in V79 cells by 2-[(aminopropyl) amino] ethanethiol under conditions of acute hypoxia. *Radiat. Res.*, **117**, 251.

GRDINA, D.J., SIGDESTAD, C.P. & CARNES, B.A. (1989*b*). Protection by WR 1065 and WR 151326 against fission-neutron-induced mutations at the HGPRT locus in V79 cells. *Radiat. Res.*, **117**, 500.

HILL, C.K., NAGY, B., PERAINO, C. & GRDINA, D.J. (1986). 2-[(aminopropyl) amino] ethanethiol (WR 1065) is anti-neoplastic and anti-mutagenic when given during $^{60}$Co gamma ray irradiation. *Carcinogenesis*, **7**, 665.

KOHN, K.W. (1979). DNA as a target in cancer chemotherapy: measurement of macromolecular DNA damage produced in mammalian cells by anticancer agents and carcinogens. *Methods Cancer Res.*, **16**, 291.

LEBKOWSKI, J.S. & LAEMMLI, U.K. (1982). Evidence for two levels of DNA folding in histone-depleted HeLa interphase nuclei. *J. Mol. Biol.*, **156**, 309.

LEPECQ, J.-B. & PAOLETTI, C. (1967). A fluorescent complex between ethidium bromide and nucleic acids: physical-chemical characterization. *J. Mol. Biol.*, **27**, 87.

MEYN, R.E. & JENKINS, W.T. (1983). Variation in normal and tumour tissue sensitivity of mice to ionising radiation-induced DNA strand breaks *in vivo*. *Cancer Res.*, **43**, 5668.

MILAS, L., HUNTER, N., STEPHENS, C.L. & PETERS, L.J. (1984). Inhibition of radiation carcinogenesis by S-2-(3-aminopropylamino)-ethylphorothioic acid. *Cancer Res.*, **46**, 1132.

MILNER, A.E., VAUGHAN, A.T.M. & CLARK, I.P. (1987). Measurement of DNA damage in mammalian cells using flow cytometry. *Radiat. Res.*, **109**, 108.

MORRISSEY, J.H. (1981). Silver stain for protein in polyacrylamide gels: a modified procedure with enhanced uniform sensitivity. *Anal. Biochem.*, **117**, 307.

PHILIPS, T.L. (1980). Rationale for initial clinical trials and future development of radioprotectors. *Cancer Clin. Trials*, **3**, 165.

SIGDESTAD, C.P., TREACY, S.H., KNAPP, L.A. & GRDINA, D.J. (1987). The effects of 2-[(aminopropyl) amino] ethanethiol (WR 1065) on radiation induced DNA double strand damage and repair in V79 cells. *Br. J. Cancer*, **55**, 477.

VOGELSTEIN, B., PARDOLL, D.M. & COFFEY, D.S. (1980). Supercoiled loops and eukaryotic DNA replication. *Cell*, **22**, 79.

YUHAS, J.M., SPELLMAN, J.M. & CULO, F. (1980). The role of WR 2721 in radiotherapy and/or chemotherapy. *Cancer Clin. Trials*, **3**, 221.